# The Combination of Low Skeletal Muscle Mass and High Tumor Interleukin-6 Associates with Decreased Survival in Clear Cell Renal Cell Carcinoma

**DOI:** 10.3390/cancers12061605

**Published:** 2020-06-17

**Authors:** Joshua K. Kays, Leonidas G. Koniaris, Caleb A. Cooper, Roberto Pili, Guanglong Jiang, Yunlong Liu, Teresa A. Zimmers

**Affiliations:** 1 Departments of Surgery, IU School of Medicine, Indianapolis, IN 46202, USA; joshkays@iupui.edu (J.K.K.); lkoniari@iu.edu (L.G.K.); Caleb.Cooper@uchospitals.edu (C.A.C.); 2 Indiana University Simon Cancer Center, Indianapolis, IN 46202, USA; rpili@buffalo.edu (R.P.); yunliu@iu.edu (Y.L.); 3 Indiana Center for Musculoskeletal Health, Indianapolis, IN 46202, USA; ggjiang@iu.edu; 4 Indiana University Purdue University Indianapolis Center for Cachexia Research Innovation and Therapy, Indianapolis, IN 46202, USA; 5 Department of Medicine, IU School of Medicine, Indianapolis, IN 46202, USA; 6 Department of Medical and Molecular Genetics, IU School of Medicine, Indianapolis, IN 46202, USA; 7 Richard L. Roudebush Veterans Administration Medical Center, Indianapolis, IN 46202, USA

**Keywords:** cachexia, body composition, kidney cancer, renal cancer, renal cell carcinoma, cytokines, body composition, muscle wasting, mortality, prognosis, risk stratification

## Abstract

Clear cell renal carcinoma (ccRCC) is frequently associated with cachexia which is itself associated with decreased survival and quality of life. We examined relationships among body phenotype, tumor gene expression, and survival. Demographic, clinical, computed tomography (CT) scans and tumor RNASeq for 217 ccRCC patients were acquired from the Cancer Imaging Archive and The Cancer Genome Atlas (TCGA). Skeletal muscle and fat masses measured from CT scans and tumor cytokine gene expression were compared with survival by univariate and multivariate analysis. Patients in the lowest skeletal muscle mass (SKM) quartile had significantly shorter overall survival versus the top three SKM quartiles. Patients who fell into the lowest quartiles for visceral adipose mass (VAT) and subcutaneous adipose mass (SCAT) also demonstrated significantly shorter overall survival. Multiple tumor cytokines correlated with mortality, most strongly interleukin-6 (IL-6); high IL-6 expression was associated with significantly decreased survival. The combination of low SKM/high IL-6 was associated with significantly lower overall survival compared to high SKM/low IL-6 expression (26.1 months vs. not reached; *p* < 0.001) and an increased risk of mortality (HR = 5.95; 95% CI = 2.86–12.38). In conclusion, tumor cytokine expression, body composition, and survival are closely related, with low SKM/high IL-6 expression portending worse prognosis in ccRCC.

## 1. Introduction

Renal Cell Carcinoma is the third most common cancer of the genitourinary tract [1] and its incidence is increasing in the United States [2]. The clear cell variant of Renal Cell Carcinoma (ccRCC) represents over 80% of all histologic subtypes of renal cancer [3], and is frequently associated with the development cachexia symptoms [4,5,6]. Cachexia, a complex metabolic derangement characterized by skeletal muscle and fat loss develops in a significant number of patients with ccRCC. Previous studies have shown that development of cachexia is associated with a three-fold increased rate of disease recurrence and a four-fold increased risk of disease specific death in patients with ccRCC [7]. 

Cachexia, which affects up to 80% of patients with advanced cancer [8], is also associated with decreased quality of life, poor response to chemotherapy, and has been implicated as the cause of death as many as 20% of cancer patients [9,10,11]. Furthermore it has been established that the development of cachexia symptoms is associated with a decreased overall survival [3,6,12,13], and poor outcomes following surgery or targeted therapy for ccRCC [14,15,16,17]. Additionally, low muscle mass has been shown to be a significant predictor of sorafenib toxicity in patients with metastatic renal carcinoma [18].

Cachexia is caused, in part, by the pleiotropic actions of inflammatory cytokines on multiple tissues from modulating the tumor microenvironment, to disrupting hematopoiesis, to altering central mechanisms of food intake, basal temperature, activity, and energy expenditure, to direct induction of lipolysis, muscle catabolism and bone loss [19,20]. The collective action of cytokines result in impaired host defenses and wasting of peripheral tissues, leading to reduced response to chemotherapy, high rates of adverse events, progressive functional decline, and death. Cytokines implicated in these disease processes, either through association in patient studies or via functional studies in animal models include Tumor Necrosis Factor (TNF), interleukin-1α (IL-1α), interferon-gamma (INF-γ), members of the Transforming Growth Factor-β (TGF-β) superfamily, including TGF-β itself as well as the Activins, Myostatin, Growth Differentiation Factor-15 (GDF-15), GDF-11, and members of the Interleukin-6/GP130 superfamily, including IL-6, Leukemia Inhibitor Factor (LIF), and ciliary neurotrophic factor (CNTF) [21,22,23] Among these, TNF [24] and IL-6 [25] are the best studied, followed by IL-1α [26], Activin [27], and TGF-β [28], with far less information about the other cytokines. 

Consistent with a role for inflammatory cytokines in cachexia of patients with ccRCC, cytokines and markers of the inflammatory response such as C-reactive protein, low albumin and high neutrophil-to-lymphocyte ratio are also associated with poor outcomes in ccRCC. Serum concentrations of TNF-∝, IL-6 and INF-γ are elevated in a subset of patients with ccRCC compared to healthy controls [29]. Elevated serum L-6 has also been shown to be a negative prognostic factor in patients with renal cell carcinoma [29,30] and to associate with weight loss and other paraneoplastic symptoms in this disease [31]. Less studied is the association of tumor-expressed cytokines and the systemic response to cancer.

This study uses a national database of patient CT scans, demographic information, tumor gene expression data and disease specific data to assesses the association between skeletal muscle (SKM), visceral adipose (VAT), and subcutaneous adipose (SCAT) mass and overall survival in patients with ccRCC. Additionally, tumor-derived cachexia-associated cytokines were assessed for correlation with skeletal muscle, fat, and overall survival. The results demonstrate, for the first time, a strong association of low muscle mass and high tumor IL-6 expression with mortality, a finding that could eventually be useful in treatment and prognostication in ccRCC.

## 2. Results

### 2.1. Patient Clinical Characteristics

The baseline patient characteristics were determined and are shown in Table 1. The overall mean age was 59.65 years (SD = 12.46) with no significant difference between men and women. There was no significant difference in tumor grade, American Joint Committee on Cancer (AJCC) stage, or tumor laterality between men and women. 

### 2.2. Body Composition

The analysis of body composition by age showed patients below the median age had significantly more SKM but no difference in VAT or SCAT when compared to those above the median (Table 2). The lower stage was also associated with increased SKM, VAT and SCAT (Table 2).

### 2.3. Characteristics Associated with Survival

**Figure 1:**
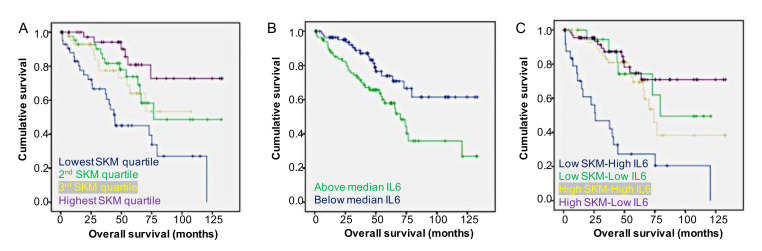
Survival stratified by skeletal muscle and IL-6 expression. (**A**) Patients in the lowest skeletal muscle mass quartile (blue line) had significantly shorter overall survival compared to the highest quartile (red line), second highest quartile (yellow line) and third highest quartile (green line). (**B**) Patients with IL-6 expression above the median (green line) have significantly shorter overall survival compared to those with expression below the median (blue line). (**C**) The combination of low SKM/high IL-6 expression (blue line) resulted in a decreased survival compared to high SKM/low IL-6 (red line), high SKM/high IL-6 (yellow line) and low SKM/low IL-6 (green line). SKM, skeletal muscle.

The median overall survival (OS) for all patients was calculated using Kaplan–Meier analysis methods and was 75.2 months. There was no sex-specific difference in overall survival, which was 75.6 months for males and 64.6 months for females (*p* = 0.123). The univariate analysis revealed that age greater than median, lowest SKM, VAT and SCAT quartiles, AJCC stage 3/4, and left sided tumors were associated with increased risk of mortality (Table 3). The Kaplan–Meier analysis showed the highest three SKM quartiles had significantly longer overall survival than the bottom quartile (Figure 1A). This was also true for IL-6 expression below the median (Figure 1B). The combination of low SKM/high IL-6 expression showed significantly worse overall survival than the combination of high SKM/low IL-6 expression (Figure 1C). 

### 2.4. Body Composition Versus Tumor Gene Expression

Body composition and tumor gene expression were analyzed for correlation, and the results are shown in Table 4. SKM was found to have significant direct correlation with INHBB and TGFB2. VAT had a significant direct correlation with INHBB and inversely correlates with CCL2. SCAT was found to have significant direct correlation with CNTF. Seven genes were found to be associated with ACM and are summarized in Table 4. Of these seven genes, two were found to have a strong inverse correlation with survival, IL-6 and IL-11. Others include INHA, OSM, IL1A, TGFB1, and CLCF1.

### 2.5. Body Composition and Tumor Gene Expression Versus Survival

Patients were then stratified into groups based on SKM mass and expression of each cytokine shown to have a significant association with survival. Of all possible combinations, low SKM/high IL-6 and low SKM/high CLCF1 demonstrated the lowest overall median survival at 26.1 months (Table 5). The combination of low SKM/high IL-6 expression had the highest risk of mortality with HR = 5.95 (95%CI = 2.86–12.38) (Table 5). 

## 3. Discussion

The current study evaluated the associations between body composition, tumor cytokine gene expression and survival in patients with ccRCC. To the authors knowledge, this is the first study to examine these relationships in ccRCC. The data show a clear relationship between body composition, tumor cytokine expression, and survival in ccRCC. The most notable relationship is the association of the combination of low SKM/high IL-6 expression and significantly decreased survival. These results provide additional evidence of the interconnectivity of these factors and support their potential as prognostic factors and possibly targets for intervention to improve outcomes. 

Sarcopenia, defined as low skeletal muscle mass, is a well-established poor prognostic factor for patients with malignancies including ccRCC [32]. One of the most widely used prognostic models for ccRCC is the Memorial Sloan Kettering Cancer Center Renal Carcinoma Risk model (MSKCC-RCC), which uses the Karnofsky performance scale as its measure of performance status. This scale relies heavily on patient self-reporting and subjective physician observations, both of which can be inconsistent. The incorporation of sarcopenia into the MSKCC-RCC model has been shown to improve its predictive accuracy [32]. Unfortunately, the current study lacked all data points to calculate the MSKCC-RCC score for patients. However, our data reinforce the importance of SKM and highlight the importance of objective measures of SKM, perhaps even to fully replace subjective measures of patient performance.

Obesity has also been shown to have prognostic value in renal cell carcinoma as it has been shown to be associated with increased survival in patients with both localized and metastatic ccRCC [33,34]. While the reason for this survival advantage is unclear, the current study reinforces the notion that fat has a protective role in ccRCC. Obesity, however, is also a known risk factor for developing RCC, specifically the clear cell variant [35,36]. This paradox may be explained by how the fat is distributed in the body, and not just its general presence. 

Fat is no longer considered an inert tissue. Adipose tissue has been shown to produce multiple inflammatory cytokines including IL-6 [37,38], and in particular, visceral adipose tissue has been shown to produce IL-6 at greater levels than subcutaneous adipose tissue [39,40]. Elevated IL-6 levels have also been shown to be associated with increased invasiveness and decreased survival in other cancers, especially colon cancer [41,42]. Not only is IL-6 frequently elevated in renal cell carcinoma [31], Table 4 shows its expression is associated with a 2.3 times increased risk of mortality. No previous studies have examined the correlations between fat compartment distribution and the risk of developing ccRCC, however with the data presented in the current study showing higher levels of SCAT to be protective while higher levels of VAT show no advantage, it is possible that the culprit behind the increased risk of ccRCC in obesity is VAT. Further studies will need to be performed to determine if this hypothesis is correct.

The combination of low SKM and high IL-6 expression was shown above to be associated with a significantly decreased overall survival. IL-6, an activator of STAT3, frequently elevated in RCC [31], is known to induce tumor progression and metastasis across tumor types [43], and is sufficient to induce skeletal muscle STAT3 activation leading to muscle wasting resulting cachexia [44,45]. Tumor-specific antineoplastic agents have also been suspected to contribute to muscle and fat-wasting observed in many cancers, however, few have been characterized. Sorafenib, a tyrosine kinase inhibitor often used to treat ccRCC, is one that has been shown to independently cause muscle and fat loss, possibly via hyperactiviation of STAT3 and ERK at the muscle [46]. Dual activation of these cachexia pathways would be expected to cause severe cachexia resulting in shorter median survival. 

Although the data presented here do not show a significant correlation between IL-6 with any specific tissue, it does show that IL-6 expression to have an exceedingly strong negative correlation with overall survival. With this data in mind, along with the previously established connections between IL-6 and cachexia, care must be taken when designing chemotherapy regimens in order to prevent possible acceleration of cachexia, especially in patients who present with low skeletal muscle mass, resulting in poorer outcomes and decreased survival.

This study does have limitations. First, there was significant difference in age in the top three SKM quartiles compared to the lowest quartile. It is not surprising that the lowest quartile for SKM would be older as muscle loss is part of the aging process. The multivariate analysis showed SKM to trend towards independent association with OS, while age showed no such evidence. In a larger cohort, the authors expect that SKM would show an independent association with OS, while age would not.

There was also a difference in AJCC stage between quartiles for SKM and VAT and AJCC strongly correlated with survival. The difference for SKM occurred between the first and the fourth quartiles. OS was significantly different between the second and fourth and third and fourth SKM quartiles as well with no difference in AJCC stage, so it can be concluded that this difference between the first and fourth quartiles was not responsible for the difference in survival between the two groups. The post-hoc analysis for the difference in AJCC stage in between the VAT quartiles was unable to identify where the difference occurred, therefore, it cannot be concluded that the difference in survival between the quartiles was due to any influence from AJCC stage. 

Twenty percent (54/271) of the patients with data available in the registry were removed from the study due to CT scans that were unable to be analyzed. This represents a significant number and inclusion of these patients may have influenced the results. Additionally, several variables of interest were not available in the dataset including all variables in the MSKCC-RCC risk model, type of chemotherapy, and BMI. 

Despite these limitations, the study demonstrates a clear and significant difference in OS based on body composition and tumor cytokine expression in ccRCC. Additionally, the data were collected from a national tumor registry, increasing the probability of a true representation of the national population and not just a specific area of the country allowing for the results to be applied broadly.

Currently, there are limited opportunities for medical oncologists and surgical oncologists to assess muscularity in patients with cancer. Few oncology clinics monitor body composition quantitatively. With the advent of automated body composition analysis from diagnostic CT or MRI scans, however, the standardized reporting of muscularity could become commonplace. Moreover, in the era of precision medicine for oncology, many centers routinely sequence tumors, both genomic and transcriptomic. Such molecular characterization of tumors, even for single features such as IL6, could also become as routine as immunohistochemistry is now. These tools would permit the stratification of patient risk of mortality, and ultimately, targeted therapy. 

## 4. Materials and Methods 

The Cancer Imaging Archive (TCIA) [47,48] and The Cancer Genome Atlas (TCGA) https://www.cancer.gov/tcga were queried for patients with ccRCC. Demographic and clinical data are available for 536 patients and RNAseq data are available for 533 patients. Abdominal computed tomography (CT) scans adequate for analysis were available for 217 of the patients with available clinical data and 215 of the patients with available RNAseq data. Images were analyzed for cross-sectional area (cm^2^) of SKM, VAT, and SCAT at the level of the third lumbar vertebrae (L3) using Slice-O-Matic software V4.3 (Tomovision, Montreal, Quebec, Canada) (Figure 1). Hounsfield unit thresholds were set at −29 to +150 for SKM, −50 to −150 for VAT, and at −30 to −190 for SCAT. Two consecutive images at L3 were assessed. The mean of the 2 images was used for statistical analysis. Total muscle measurements included the rectus abdominus, external and internal oblique, transversus abdominus, psoas, erector spinae, and quadratus lumborum.

The TCGA database was queried for RNAseq data for the 217 patients with analyzed CT scans. Only 2 patients did not have RNAseq data available resulting in a total of 215 patients. A total of 20,531 total genes were identified. A total of 1,369 genes were found to be expressed in <10% of the samples and were removed resulting in 19,162 genes for analysis.

Patients were divided into quartile for each tissue by sex. The male and female quartiles were analyzed individually and combined by the Kaplan–Meier method to evaluate for differences in overall survival. When a difference was present, Kaplan–Meier survival curves were created between each quartile to identify where the difference existed. Univariate and multivariate Cox proportional hazard models were created for each sex and the sexes combined. All hazard ratios are reported 95% confidence intervals and *p* values. 

Body composition and overall survival were compared with tumor gene expression of 21 cachexia-associated cytokines which included Interleukin-1 alpha (IL1a), Interleukin-1 beta (IL1b), Interleukin-6 (IL6), Interleukin-11 (IL11), Tumor Necrosis Factor (TNF), Growth Differentiation Factor-11 (GDF11), Growth Differentiation Factor-15 (GDF15), Myostatin (MSTN), Oncostatin M (OSM), Chemokine C-C motif ligand 2 (CCL2), Ciliary Neurotrophic Factor (CNTF), Cardiotrophin-like Cytokine Factor 1 (CLCF1), Cardiotrophin 1 (CTF1), Inhibin alpha (INHA), Inhibin Beta A (INHBA), Inhbin Beta B (INHBB), Follistatin (FST), Follistatin-like 3 (FSTL3), Transforming Growth Factor Beta-1 (TGFB1), Transforming Growth Factor Beta-2 (TGFB2), and Transforming Growth Factor Beta-3 (TGFB3). Spearman’s rank correlation with false discovery rate for multiple comparisons was performed. A rho value >0.1 and *p* value < 0.05 were considered significant. Only significant associations are reported. 

Continuous variables were assessed with student t-test or one-way analysis of variance (ANOVA) when appropriate. Categorical variables were analyzed using chi-squared test and Mann–Whitney U test. All results are reported as means with standard deviation. All statistical analyses were completed using IBM SPSS Statistics for Windows version 23.0 (SPSS, Chicago, IL). Results were considered significant at the *p* < 0.05. 

## 5. Conclusions

Low skeletal muscle and visceral and subcutaneous adipose masses have a clear and significant association with decreased overall survival in patients with clear cell renal carcinoma. These findings support previous studies, agreeing that cachexia is a major cause of mortality in cancer, adding ccRCC to the list of malignancies in which this association has been made. Additionally, a number of tumor-derived cytokines are associated with an increased risk of all-cause mortality, perhaps most importantly IL-6. Finally, we show, for the first time, that the combination of low skeletal muscle mass and high IL-6 expression is an especially concerning combination that predicts early death in patients with ccRCC. Ultimately, once the appropriate tools are available, including automated body composition analysis and reporting of tumor transcriptomics, these factors could be considered when designing a patient care plan and counseling ccRCC patients on prognoses.

## Figures and Tables

**Table 1:** Patient Characteristics *n* = 217.

Variable	No. (%)	Male/Female	*p* Value
Age, mean years (SD)	59.7 (12.5)		0.09
Males	58.6 (12.6)		
Females	61.6 (12.1)		
Tumor Grade			
Grade 1	0 (0)	0/0	
Grade 2	87 (40)	48/39	
Grade 3	94 (43)	69/25	0.123
Grade 4	36 (17)	22/14	
AJCC Stage			
Stage 1	112 (52)	71/41	
Stage 2	18 (8)	17/1	
Stage 3	54 (25)	34/20	0.459
Stage 4	33 (15)	17/16	
Tumor Laterality			
Right	116 (53)	78/38	0.295
Left	101 (47)	61/40	

Abbreviations: AJCC = American Joint Commission on Cancer.

**Table 2:** Body Composition/Tissue Area Measurements.

Variation	Mean SKM, cm^2^ (SD)	*p* Value	Mean VAT, cm^2^ (SD)	*p* Value	Mean SCAT, cm^2^ (SD)	*p* Value
Overall	155.1 (41.3)		179.3 (109.9)		200.8 (93.3)	
Top Quartile	192.3 (38.1)	<0.001	310.0 (85.3)	<0.001	326.6 (73.3)	<0.001
Bottom Quartile	121.9 (28.6)	<0.001	65.2 (41.8)	<0.001	104.5 (27.0)	<0.001
Age						
<59.7 years	169.3 (41.3)	<0.001	177.4 (107.8)	0.80	205.4 (94.5)	0.49
≥59.7 years	140.1 (35.6)		181.2 (112.5)		195.5 (92.1)	
AJCC Stage						
Stage I	159.8 (42.3)	<0.001	193.6 (109.7)	<0.001	212.3 (99.0)	0.16
Stage II	181.5 (37.6)		247.9 (143.4)		204.4 (75.3)	
Stage III	149.4 (39.3)		165.2 (143.4)		197.3 (93.4)	
Stage IV	133.7 (31.2)		116.0 (71.0)		166.2 (77.1)	

Abbreviations: SKM = skeletal muscle mass, VAT = visceral adipose tissue mass, SCAT = subcutaneous adipose tissue mass.

**Table 3:** Association with All-Cause Mortality.

	Univariate Analysis		Multivariate Analysis	
Variation	HR (95% CI)	*p* Value	HR (95% CI)	*p* Value
Overall				
Age				
<59.7 years	Ref	0.004	Ref	0.440
≥59.7 years	2.1 (1.3–3.6)		1.3 (0.7–2.6)	
SKM mass				
Top 3 quartiles	Ref	<0.001	Ref	0.081
Lowest quartile	3.2 (2.0–5.3)		1.8 (0.9–3.5)	
VAT mass				
Top 3 quartiles	Ref	0.03	Ref	0.61
Lowest quartile	1.8 (1.1–3.0)		1.2 (0.6–2.6)	
SCAT mass				
Top 3 quartiles	Ref	<0.001	Ref	0.027
Lowest quartile	2.7 (1.6–4.7)		2.4 (1.1–5.4)	
Tumor Grade				
Grade 1/2	Ref	0.067	Ref	0.10
Grade 3/4	1.7 (0.96–2.9)		0.52 (0.2–1.1)	
AJCC Stage				
Stage 1/2	Ref	<0.001	Ref	<0.001
Stage 3/4	4.0 (2.4–6.8)		7.0 (3.3–14.5)	
Laterality				
Right	Ref	0.023	Ref	0.004
Left	1.8 (1.1–2.9)		2.3 (1.3–4.2)	
Sex				
Male	Ref	0.123	Not Included	
Female	1.5 (0.9–2.3)			

Abbreviations: SKM = skeletal muscle mass, VAT = visceral adipose tissue mass, SCAT = subcutaneous adipose tissue mass, AJCC = American Joint Commission on Cancer.

**Table 4:** Tumor Gene Expression and Associations with Body Composition and Overall Survival.

Tissue	Gene	Correlation Coefficient	*p* Value
SKM	INHBB TGFB_2_	0.149 0.150	0.029 0.028
VAT	INHBB CCL2	1.79 −0.172	0.009 0.012
SCAT	CNTF	0.167	0.027
**Gene**	**Correlation Coefficient**	**Hazard Ratio**	***p* Value**
IL6	0.835	2.31	<0.0001
IL11	0.700	2.01	<0.0001
INHA	0.622	1.86	0.0001
OSM	0.585	1.80	0.0004
IL1A	0.483	1.62	0.003
TGFB1	0.422	1.52	0.008
CLCF1	0.400	1.49	0.01

Abbreviations: SKM = skeletal muscle mass, VAT = visceral adipose tissue mass, SCAT = subcutaneous adipose tissue mass, INHBB = Inhibin beta B, TGFB_2_ = Transforming Growth Factor β_2_, IL11 = Interluekin-11, GDF11 = growth differentiation factor 11, CCL2 = C-C Motif Chemokine Ligand 2, CNTF = Ciliary Neurotrophic Factor, IL6 = Interleukin 6. IL11 = Interleukin 11. INHA = Inhibin-α. OSM = Oncostatin M. IL1A = Interluekin 1a. TGFB1 = Transforming Growth Factor β1. CLCF1 = Cardiotrophin-like Cytokine Factor 1.

**Table 5:** Skeletal Muscle Mass-Cytokine Expression Survival and Mortality Risk.

Group	Median OS (Months)	*p* Value	Hazard Ratio	95% CI
SKM/IL6				
High SKM/Low IL6	Not Defined ^a^	<0.001	Reference	
High SKM/High IL6	79.5		1.67	0.81–3.45
Low SKM/Low IL6	74.2		1.45	0.54–3.87
Low SKM/High IL6	26.1		5.95	2.86–12.38
SKM/INHA				
High SKM/Low INHA	Not Defined ^a^	<0.001	Reference	
High SKM/High INHA	70.2		1.82	0.88–3.77
Low SKM/Low INHA	Not Defined ^a^		3.17	1.24–8.10
Low SKM/High INHA	40.00		4.71	2.24–9.87
SKM/IL11				
High SKM/Low IL11	Not Defined ^a^	<0.001	Reference	
High SKM/High IL11	Not Defined ^a^		1.39	0.68–2.84
Low SKM/Low IL11	73.0		2.54	1.08–5.97
Low SKM/High IL11	40.0		4.71	2.24–9.94
SKM/OSM				
High SKM/Low OSM	Not Defined ^a^	<0.001	Reference	
High SKM/High OSM	Not Defined ^a^		0.83	0.41–1.67
Low SKM/Low OSM	79.5		1.45	0.57–3.71
Low SKM/High OSM	37.8		4.10	2.08–8.06
SKM/IL1A				
High SKM/Low IL1A	Not Defined ^a^	0.001	Reference	
High SKM/High IL1A	Not Defined ^a^		0.93	0.47–1.87
Low SKM/Low IL1A	75.2		2.40	0.99–5.85
Low SKM/High IL1A	43.9		2.84	1.42–5.68
SKM/TGFB1				
High SKM/Low TGFB1	Not Defined ^a^	<0.001	Reference	
High SKM/High TGFB1	Not Defined ^a^		1.31	0.65–2.66
Low SKM/Low TGFB1	41.0		3.95	1.74–8.93
Low SKM/High TGFB1	44.6		3.15	1.50–6.60
SKM/CLCF1				
High SKM/Low CLCF1	Not Defined ^a^	<0.001	Reference	
High SKM/High CLCF1	Not Defined ^a^		0.53	0.26–1.09
Low SKM/Low CLCF1	75.2		1.44	0.65–3.18
Low SKM/High CLCF1	26.1		3.44	1.74–6.82

Abbreviations: SKM—skeletal muscle mass; OS—overall survival; IL-6—interleukin-6; INHA—inhibin alpha; IL-11—interleukin-11; OSM—oncostatin M; IL1A—interleukin 1 alpha; TGFB1 – tumor growth factor beta 1; CLCF1 —cardiotrophin like cytokine factor 1. ^a^—median survival was not reached as greater than 50% of patients were still alive at time of analysis.
